# Elucidating Gas Reduction Effects of Organosilicon Additives in Lithium-Ion Batteries

**DOI:** 10.1021/jacs.5c00402

**Published:** 2025-02-26

**Authors:** Jingyang Wang, Sarah L. Guillot, Monica L. Usrey, Tingzheng Hou, Kristin A. Persson

**Affiliations:** a Materials Sciences Division, Lawrence Berkeley National Laboratory, Berkeley, California 94720, United States; b Silatronix, Inc., Madison, Wisconsin 53704, United States; c Department of Materials Science and Engineering, University of California Berkeley, 210 Hearst Mining Building, Berkeley, California 94720, United States; d Institute of Materials Research, Tsinghua Shenzhen International Graduate School, Tsinghua University, Shenzhen, Guangdong 518055, China

## Abstract

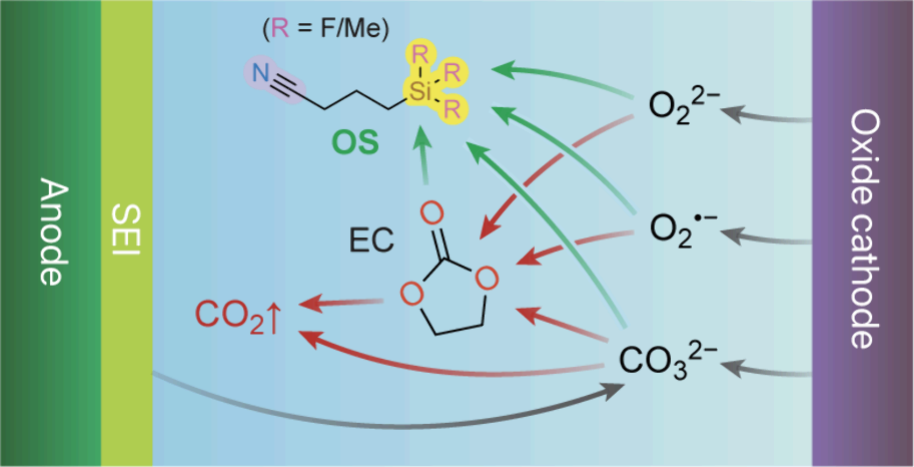

Lithium-ion batteries (LIBs) with nonaqueous liquid electrolytes are prone to gas generation at elevated voltages and temperatures, degrading battery performance and posing serious safety risks. Organosilicon (OS) additives are an emerging candidate solution for gassing problems in LIBs, but a detailed understanding of their functional mechanisms remains elusive. In this work, we present a combined computational and experimental study to elucidate the gas-reducing effects of OS additives. Cell volume measurements and gas chromatography–mass spectrometry reveal that OS additives can substantially reduce gas evolution in LIBs, particularly CO_2_ regardless of source. Through density functional theory calculations, we identify multiple plausible pathways for CO_2_ evolution, including (1) nucleophile-induced ring-opening of ethylene carbonate (EC) and the subsequent electro-oxidation and (2) direct electro-oxidation of lithium carbonate (Li_2_CO_3_). Correspondingly, we find that OS additives function via two primary mechanisms: (1) scavenging of nucleophiles such as superoxide (O_2_^•–^), peroxide (O_2_^2–^), and carbonate ion (CO_3_^2–^); (2) oligomerization with ethylene carbonate oxide ion and ethylene dicarbonate ion. Moreover, we discover that OS additives possess strong lithium coordination affinity, which helps further reduce the nucleophilic reaction energies and hence increases their nucleophile-scavenging efficiency. Finally, we provide a mechanistic interpretation for the enhanced gas-reduction effects observed with fluorinated OS compounds, corroborated by surface analysis results from X-ray photoelectron spectroscopy. Our study offers the first molecular-level insights into how OS additives contribute to reduced gas formation in LIBs, paving the way for improved safety and performance of LIBs.

## Introduction

Nonaqueous liquid electrolytes constitute a major component of modern lithium-ion battery (LIB) technologies.^1−4^ The performance of LIBs critically depends upon several key properties of the electrolytes, including reduction/oxidation stability, thermal stability, and ionic conductivity.^5^ For a given electrolyte component, its susceptibility to (electro)chemical decomposition directly impacts the battery’s Coulombic efficiency, as well as the growth and chemical composition of the passivation layers formed on electrodes.^6^ The decomposition rate of common solvents, such as carbonates and ethers, accelerates at elevated voltages and temperatures,^7−10^ posing a significant challenge to maintaining long-term capacity in high-voltage LIBs. Furthermore, continuous solvent decomposition is a primary contributor of gaseous byproducts such as carbon dioxide (CO_2_), carbon monoxide (CO), ethylene (C_2_H_4_), etc., resulting in battery capacity degradation and even inducing serious safety risks to the normal operation of battery cells.^11−18^

In recent years, these pressing challenges have led to the exploration of a wide variety of potential remedies, including electrode surface coatings,^14,19^ salt substitution,^17^ surface composition and morphology modifications,^16^ and electrolyte additives.^20^ Among these options, small-molecule functional additives have been garnering significant attention as one of the most cost-effective methods to enhance battery durability without sacrificing its performance.^21^ These additives typically function by intervening in the decomposition pathways of electrolytes and forming nonreactive electrolyte-protective products.^22^ The effectiveness of additives is directly tied to their electron-donating or electron-accepting properties.^6^ Experimental evidence suggests that even a small amount of additives can effectively inhibit the continuous decomposition of organic solvents without compromising the electrolyte’s transport properties.^23−25^

**Figure 1:**
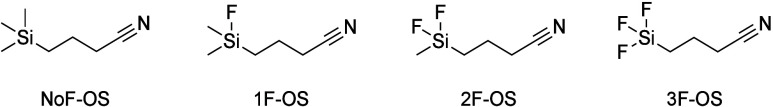
Molecular structures of the organosilicon (OS) nitrile additives considered in this work. Degrees of fluorination in the silyl group are labeled nonfluorinated (NoF), monofluorinated (1F), difluorinated (2F), and trifluorinated (3F), respectively.

A LIB electrolyte additive’s chemical composition and structure critically affect its functional performance. Commonly employed functional additives include vinylene carbonate (VC), fluoroethylene carbonate (FEC), succinonitrile (SN), 1,3-propane sultone (PS), and tris(trimethylsilyl) phosphate (TMSP).^26−28^ In recent years, organosilicon compounds have been proposed as ideal candidate additives for high-energy-density LIBs due to their high thermal and electrochemical stability, low flammability, and environmental friendliness.^29−33^ In particular, Guillot et al.^34^ showed that certain organosilicon (OS) additives with Li^+^-coordinating functional groups (e.g., cyano group) (Figure 1) drastically reduce various gaseous products at a higher normalized activity than that of SN and PS. Notably, they showed that a mere 3 vol % OS can eliminate 94–98% of CO_2_ generated from high-temperature (60 °C) storage of NMC622/Gr pouch cells with various carbonate-based electrolytes. Interestingly, the extent of gas suppression was observed to increase with a higher degree of fluorination in the silyl group of the OS compounds.^34,35^

While the exceptional effectiveness of the OS additives in LIB gas reduction has been experimentally established, the mechanistic origin of their superior performance is still subject to active debate. Several mechanistic hypotheses have been proposed, including (1) formation of passivating interfacial layers, (2) scavenging of reactive oxygen species released from the cathode at high voltages,^35^ and (3) reactions with soluble solid electrolyte interphase (SEI) components migrated to the cathode, or with native cathode surface impurities such as lithium carbonate (Li_2_CO_3_).^34^ Several experimental attempts involving OS additives under storage or controlled conditions exist,^34,35^ yet none of them provided a comprehensive analysis of molecular reaction pathways of OS under working conditions of LIBs. Understanding the exact mechanisms of this process would pave the way for designing and optimizing future battery electrolyte additives.

In this work, we present a combined computational and experimental study to investigate the role of organosilicon additives in liquid Li-ion battery electrolytes. First, we utilize cell volume measurements and gas chromatography–mass spectrometry (GC-MS) to illustrate the gas-reducing behavior of OS additives, providing the basis for the subsequent theoretical analyses. Using classical molecular dynamics (MD) simulations, we elucidate the influence of organosilicon additives on the solvation structure of electrolytes and its impact on the electrolyte ionic conductivity. Next, through density functional theory (DFT) calculations, we identify energetically favorable oxidative pathways for the generation of CO_2_, a major gaseous product, from ethylene carbonate (EC) and lithium carbonate (Li_2_CO_3_). Finally, we reveal that chemical oxidation pathways, rather than electrochemical ones, are the primary mechanism behind CO_2_ suppression by OS additives, providing insights into the gas-reducing functionality of these compounds.

## Results and Discussion

### Experimental Measurements of Gas Reduction

**Figure 2:**
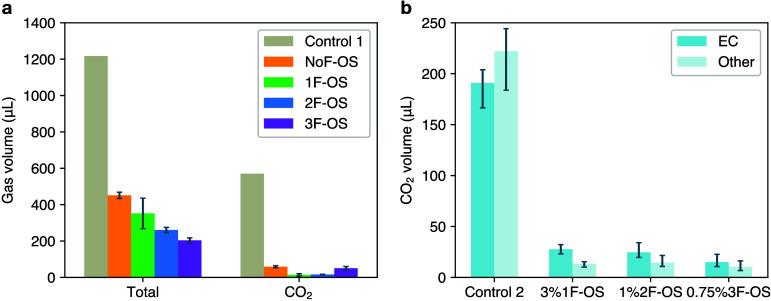
(a) Gas volume increase after 4 weeks of storage at 60 °C relative to after formation in 4.3 V SC-NMC811/Gr pouch cells with control 1 and 3% NoF-OS/1F-OS/2F-OS/3F-OS electrolytes. (b) CO_2_ volume in a separate experiment (also 4 weeks storage at 60 °C in 4.3 V SC-NMC811/Gr pouch cells) with control 2 and 3% 1F-OS/1% 2F-OS/0.75% 3F-OS electrolytes, with ^13^C-labeled EC and GC-MS employed to quantify CO_2_ originating from EC (^13^CO_2_, *m*/*z* 45) versus other sources (^12^CO_2_, *m*/*z* 44).

In this study, we choose single crystal LiNi_0.8_Mn_0.1_Co_0.1_O_2_/graphite (SC-NMC811/Gr) pouch cells as a representative system for gassing behavior in commercial lithium-ion batteries. The control electrolyte is a solution of 1 M lithium hexafluorophosphate (LiPF_6_) in a mixture of ethylene carbonate (EC), diethyl carbonate (DEC), and ethyl methyl carbonate (EMC) (1/1/1 by volume) plus 0.5% vinylene carbonate (VC). Figure 2a shows the results of gas volume measurements of aged cells through storage at 60 °C for 4 weeks. Compared with the controlled electrolyte, the total increased gas volume of the electrolytes mixed with 3% NoF-OS, 1F-OS, 2F-OS, and 3F-OS decreased by 64%, 76%, 81%, and 84%, respectively. In particular, CO_2_ constitutes the most significant portion (47%) of all the gas species generated in the control electrolyte (Figure S1). On the other hand, the volumes of CO_2_ are found to decrease by 89%, 98%, 97%, and 91% for the respective additive-mixed electrolytes, representing the greatest percentage of reduction among all the gas species generated. This result further confirms the general effectiveness of gas reduction, especially CO_2_, by OS additives in LIBs.

In a separate set of experiments, the sources of CO_2_ generated were traced by labeling EC with ^13^C. Employing gas chromatography–mass spectrometry (GC-MS), we find that EC-generated CO_2_ accounts for nearly half (46%) of all the CO_2_ generated. (Figure 2b) This observation is qualitatively consistent with previous works suggesting that the oxidative decomposition of cyclic carbonates is the primary source of CO_2_ in LIBs with NMC cathodes.^36−39^ For electrolytes mixed with 3% 1F-OS, 1% 2F-OS, and 0.75% 3F-OS, we observe that the CO_2_ generated from EC is reduced respectively by 86%, 87%, and 92% compared to control. The CO_2_ generated from non-EC sources is reduced by an even greater amount (94%, 94%, and 95%, respectively). These results strongly imply that OS additives act uniformly on various CO_2_ sources. More importantly, combined with the previous data on the total gas volume, it becomes evident that higher-fluorinated OS compounds are likely to perform better in reducing CO_2_ generation in LIBs.

### Solvation Structure and Transport Properties

**Figure 3:**
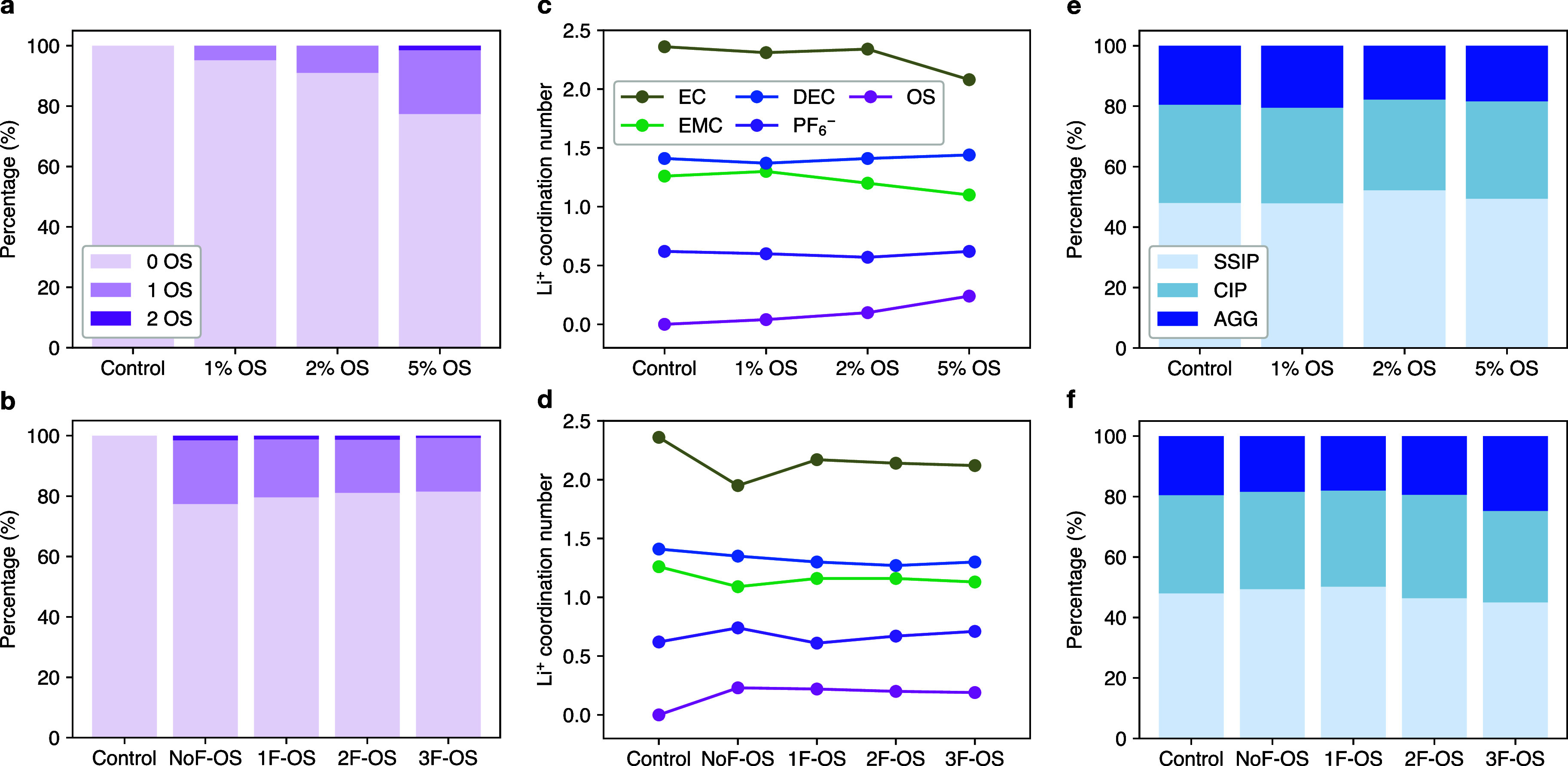
(a, b) Statistics of OS in Li^+^ first solvation shells, (c, d) average Li^+^ coordination numbers of various species, and (e, f) statistics of Li^+^ with different anion coordination states in 1 M LiPF_6_ 1:1:1 (%vol) EC:EMC:DEC solutions. (a, c, e) Control and 1/2/5% NoF-OS electrolytes; (b, d, f) control and with 5% NoF-OS/1F-OS/2F-OS/3F-OS electrolytes.

Classical molecular dynamics (MD) simulations were conducted to evaluate the solvation and transport properties of electrolytes with and without OS additives. The simulated solutions consist of 145 LiPF_6_, 685 EC, 377 DEC, and 438 EMC molecules, along with 0/8/16/40 OS molecules, corresponding to the experimental additive concentrations of 0/1/2/5%, respectively. The averaged statistics of the first solvation shell of Li^+^ are shown in Figure 3a. As the concentration of OS increases, the Li^+^ coordination number of OS rises monotonically. Notably, at a 5% concentration, NoF-OS exhibits a Li coordination number of 0.24, corresponding to 87% of all the OS molecules present. These results indicate that OS additives have a high affinity toward Li^+^. This trend is further supported by the radial distribution functions (RDFs) in Figure S2, which show that OS has the highest probability of presence near Li^+^ among all the species present. The analyses show that OS’s strong Li^+^-coordinating ability is attributed to the cyano group, while fluorine plays a negligible role in Li^+^ coordination. As shown in Figure 3c, the coordination number of OS increases with OS concentration, with the additive primarily substituting EC and EMC in the Li^+^ solvation shell. Specifically, in the 5% NoF-OS electrolyte, the average coordination number of OS is 0.24, while that of EC decreases by 0.28, from 2.36 in the control to 2.08 with 5% OS. This trend aligns with our previous findings that EC is preferentially replaced by substituting species, while the linear carbonates generally exhibit higher binding stability with Li^+^.^40,41^ Additionally, using higher-fluorinated OS at a fixed concentration leads to a minor decrease in OS’s Li^+^ coordination number (Figure 3b,d). This reduction is due to a slight decrease in the partial charge of the cyano-N atom (Table S1). These trends align with the Li^+^ binding energies calculated using DFT (Table S2).

An ion clustering analysis was conducted using the MD trajectories to evaluate further the influence of OS additives on solvation (Figure 3e,f). Ionic solvation structures were classified into solvent-separated ion pairs (SSIP), contact ion pairs (CIP), and aggregates (AGG) based on the coordination state between Li^+^ and anions. As the concentration of OS increased, no significant changes were observed in the distribution of solvation structures, indicating that the primary effect of OS is to substitute EC in the first solvation shell rather than altering the relative populations of SSIP, CIP, and AGG. However, for highly fluorinated OS species, a slight increase in the degree of ion association was observed.

The ionic conductivities calculated from the MD trajectories are shown in Figure S3. The experimental ionic conductivity for LiPF_6_ in EC:DMC is reported to be 8–10 mS cm^–1^.^1^ The calculation error is less than one order of magnitude, which is within an acceptable range for nonpolarizable force fields as they tend to underestimate atomic diffusivities.^42^ With the gradual addition of NoF-OS, the model electrolyte’s ionic conductivity remains essentially constant at ∼1.81 mS cm^–1^ with less than 3% OS, then decreases by 34% to 1.19 mS cm^–1^ with 5% OS. This reduction is primarily attributed to OS’s higher viscosity. Furthermore, at a fixed OS concentration, the ionic conductivity moderately declines with a higher degree of OS fluorination (1.07/1.00/0.90/0.83 mS cm^–1^ for 5% NoF-OS/1F-OS/2F-OS/3F-OS). As OS additives are typically applied in limited concentrations (<3%) in commercial LIB electrolytes, their negative influence on ionic conductivity should be inconsequential.

### CO_2_ Evolution Mechanisms in LIB Electrolytes

To investigate the gas-reducing role of OS additives, it is essential first to understand the molecular mechanism behind gas evolution in LIB electrolytes. In this study, we focused on CO_2_ as a representative species due to the experimental observations that CO_2_ constitutes the majority of the gas species generated from Ni-rich ternary oxide cathodes,^12,36,38,43−47^ and that OS additives are particularly effective at reducing CO_2_ compared to other gaseous byproducts^34^ (Figure S1).

Experiments have identified the oxidation of cyclic carbonate solvents, particularly EC, as the most significant source of CO_2_ in LIB electrolytes.^36,45^ Multiple pathways for CO_2_ generation from EC have been proposed in the literature.^15,38,48,49^ Importantly, previous density functional theory (DFT) calculations found that EC and its related complexes (EC-EC, EC-PF_6_^–^) are unlikely to be electro-oxidized within the normal operating voltage range of NMC811 cathodes (≤4.4 V), as they exhibit significantly higher oxidation potentials. (EC: 6.9 V; EC-EC: 5.0–5.2 V; EC-PF_6_^–^: 6.2 V in an implicit solvent with dielectric constant ε = 20.7)^50^ On the other hand, recent works by Bryantsev et al.^51^ and Spotte-Smith et al.^52^ identified an initial oxidation pathway for EC that proceeds via bimolecular nucleophilic substitution (S_N_2) at an ethylene carbon, leading to EC ring-opening via C–O bond cleavage. The latter work found that this S_N_2@C reaction is kinetically favorable when superoxide (O_2_^•–^) or peroxide (O_2_^2–^) acts as the nucleophile, while singlet oxygen significantly hinders the reaction kinetics.

**Figure 4:**
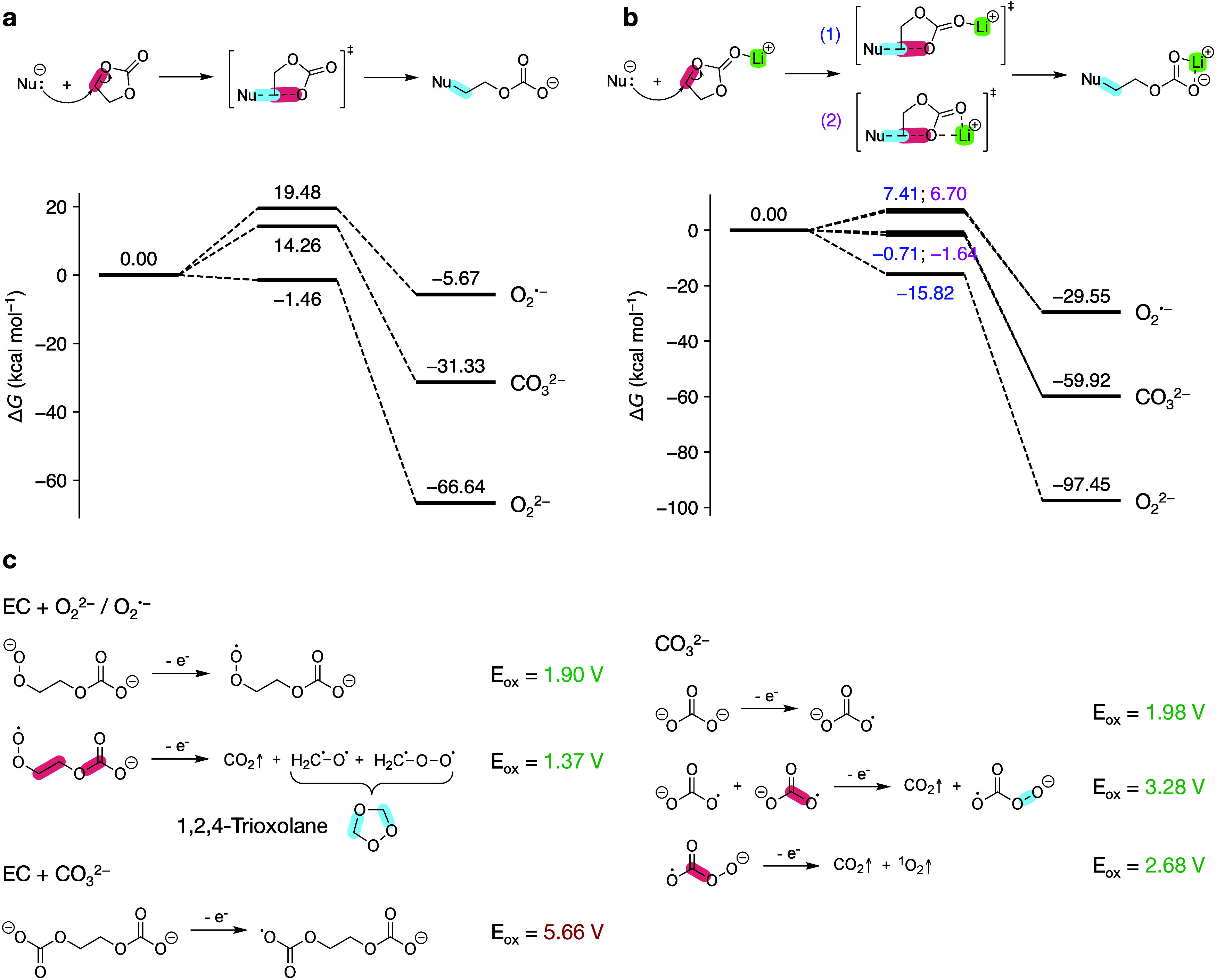
(a, b) Free energy profiles of S_N_2-type ring-opening reactions of (a) noncoordinated EC and (b) Li^+^-coordinated EC via nucleophilic substitution (superoxide, peroxide, carbonate ion) at an ethylene carbon. (1) and (2) in (b) refer to the monodentate and bidentate conformers of the transition state/intermediate state structures. Red and blue highlights indicate bond breaking and formation, respectively. (c) Electrochemical oxidation pathways of ethylene carbonate oxide, ethylene dicarbonate, and carbonate ion. Oxidation potentials are referenced to the Li^+^/Li redox couple.

We have thus recalculated the energy profiles for the EC ring-opening reactions with O_2_^•–^ and O_2_^2–^, as shown in Figure 4a. (Optimized geometries are shown in Figure S4.) The calculated relative free energies are in good agreement with the values in the literature. In particular, the reaction free energies Δ*G* are less than zero, and the free energies of activation Δ*G*^‡^ are less than 20 kcal mol**^–1^**, indicating that these pathways are both thermodynamically and kinetically favorable. Moreover, we considered the carbonate ion (CO_3_^2**–**^) as a relevant nucleophile, as experimental evidence suggests that soluble carbonate species (e.g., lithium ethyl carbonate (LEC), lithium methyl carbonate (LMC), lithium ethylene monocarbonate (LEMC), and lithium ethylene dicarbonate (LEDC)),^53,54^ generated via EC reduction on the anode side,^55−57^ can migrate from the SEI to the cathode side.^38,58,59^ The free energy profile for the EC+CO_3_^2–^ reaction is located below that for EC+O_2_^•–^ but above that for EC+O_2_^2–^, suggesting the nucleophilicity order O_2_^•–^ < CO_3_^2–^ < O_2_^2–^. In contrast, the same reaction mechanism involving the oxidized carbonate radical ion (CO_3_^•–^) is kinetically unfavorable, with Δ*G*^‡^ = 36.90 kcal mol**^–1^**. Informed by our MD simulation results, which indicate that EC preferentially binds with Li^+^ at the carbonyl oxygen site, we performed additional calculations for the same S_N_2@C reactions with Li^+^-coordinated EC (Figure 4b). Both Δ*G* and Δ*G*^‡^ are lowered compared to those of noncoordinated EC due to the increased electrophilicity of Li^+^-EC. These results strongly suggest that the rates of the initial chemical oxidation step for EC are further increased in the LIB electrolyte solvation environment.

These initial chemical oxidation pathways produce ethylene carbonate oxide and ethylene dicarbonate, which may be prone to subsequent decomposition through various chemical and electrochemical pathways, ultimately leading to gas evolution. Here, we focused on the likelihood for these products to undergo electrochemical oxidation. As shown in Figure 4c, ethylene carbonate oxide is prone to one-electron electrochemical oxidation at 1.90 V vs Li^+^/Li, which is lower than the cutoff voltage for most cathodes. A combined two-electron pathway leads to the breakdown of the molecule into CO_2_, ^•^CH_2_O^•^, and ^•^CH_2_O_2_^•^, with the latter two spontaneously recombining to form 1,2,4-trioxolane. In contrast, ethylene dicarbonate exhibits a significantly higher oxidation potential of 5.66 V vs Li^+^/Li, indicating that it is more likely to decompose via chemical pathways than electrochemical oxidation.

The oxidation of EC with reactive oxygen species can only occur at high voltages (>4.3 V) due to the high onset voltage for oxygen release from the cathode lattice. At lower voltages (∼4.1–4.2 V), another major source of CO_2_ was speculated to be the carbonates originating either from cathode surface impurities (e.g., Li_2_CO_3_) or dissolved SEI components. Experiments have shown that the oxidation potential of Li_2_CO_3_ is 3.8 V vs Li^+^/Li.^60^ Here, one possible electrochemical oxidation pathway is illustrated in Figure 4c. The overall reaction involves a four-electron process that converts two carbonate ions into two CO_2_ molecules and one singlet oxygen molecule. Additionally, a chemical decomposition pathway for Li_2_CO_3_ via reaction with POF_3_ is energetically favorable,^61^ implying that multiple mechanisms could be responsible for gas generation.

### Electrochemical Stability of OS Additives and Related Complexes

**Figure 5:**
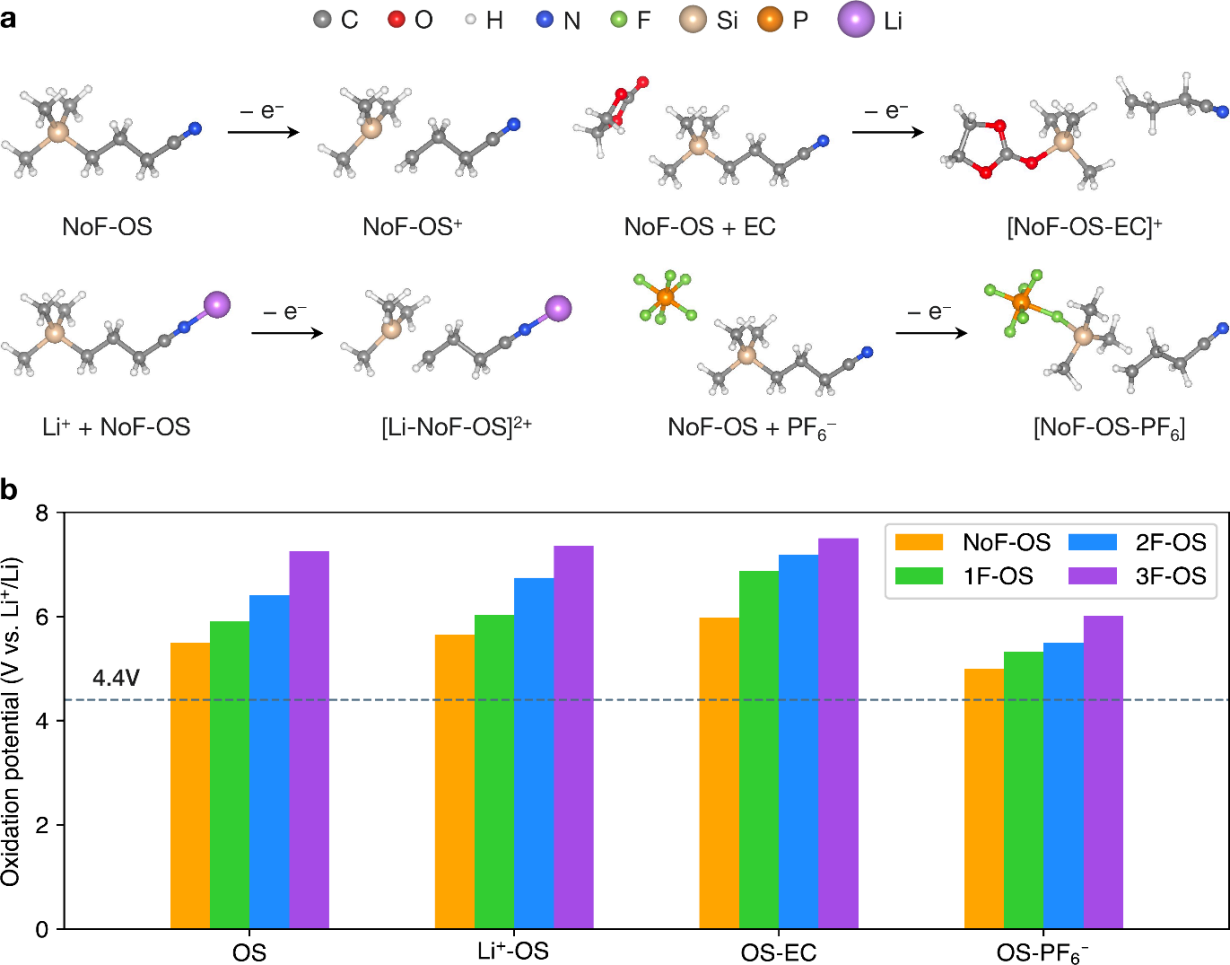
(a) Optimized geometries of NoF-OS, Li^+^-NoF-OS, NoF-OS-EC, and NoF-OS-PF_6_^–^ in their native and oxidized states. (b) Oxidation potentials of the aforementioned species, referenced to the Li^+^/Li redox couple. The horizontal line at 4.4 V vs Li^+^/Li is a typical upper voltage limit for NMC811 cathodes under normal working conditions.

To evaluate the electrochemical stability of additives and their related complexes under oxidative conditions, we performed DFT geometry optimization for these species in their natural and oxidized states. Figure 5a shows the geometry-optimized structures of four representative species in an LIB electrolyte environment: NoF-OS, Li^+^-NoF-OS, NoF-OS-EC, and NoF-OS-PF_6_^–^. Upon removal of a single electron, these species all undergo significant structural distortion. Specifically, the bond between silicon and the methylene carbon breaks as a result of oxidation of the methylene carbon atom, leaving a Me_3_Si^•^ radical. Similar behavior is observed for the fluorinated OS compounds when nucleophilic species such as the carbonyl oxygen in EC or fluorine in PF_6_^–^ are in proximity to Si (Figure S5). In these cases, a new bond forms between the nucleophile and Si, driven by the preference of Si to satisfy the octet rule.

The oxidation potentials of OS additives and their related complexes are shown in Figure 5b. Among these species, OS-PF_6_**^–^** exhibits the lowest oxidation potentials, whereas OS-EC exhibits the highest. Notably, the oxidation potentials of these species all exceed 5.0 V vs Li^+^/Li, which is 0.6 V higher than a typical “safe” upper voltage limit for NMC811 cathodes under working conditions.^15,62^ Moreover, the oxidation potential increases with a higher degree of OS fluorination, suggesting that higher-fluorinated OS and their associated complexes are even less likely to decompose into reactive species via direct oxidation. These findings provide clear evidence of the electrochemical stability of OS additives in the oxidative environment of LIB electrolytes. Furthermore, the results preclude direct electrochemical oxidation as a viable explanation of gas reduction.

### Chemical Reaction Pathways of CO_2_ Suppression by OS Additives

Based on the CO_2_ gassing mechanisms discussed earlier, we have identified the primary CO_2_-inducing agents in LIB electrolytes as anionic oxygen species (superoxide, peroxide) at high voltages (>4.3 V) and carbonate ions at lower voltages (∼4.1–4.2 V). Consequently, mechanisms of CO_2_ suppression by OS additives are likely to involve reactions with these agents. In an OS molecule, two primary reactive sites exist: silyl group and cyano group. The silyl-Si in OS additives, analogous to its carbon counterpart, is susceptible to nucleophilic substitution/addition due to its relatively low-energy vacant 3d orbital. In these reactions, the qualitative characteristics of the free energy profile are sensitive to the specific nature of the silyl group as well as the nucleophile.^63,64^ Meanwhile, it is well established that the cyano-C is prone to nucleophilic addition.^65^ Thus, we consider nucleophilic attacks by the gas-inducing agents at both the silyl-Si and cyano-C sites of OS additives.

**Figure 6:**
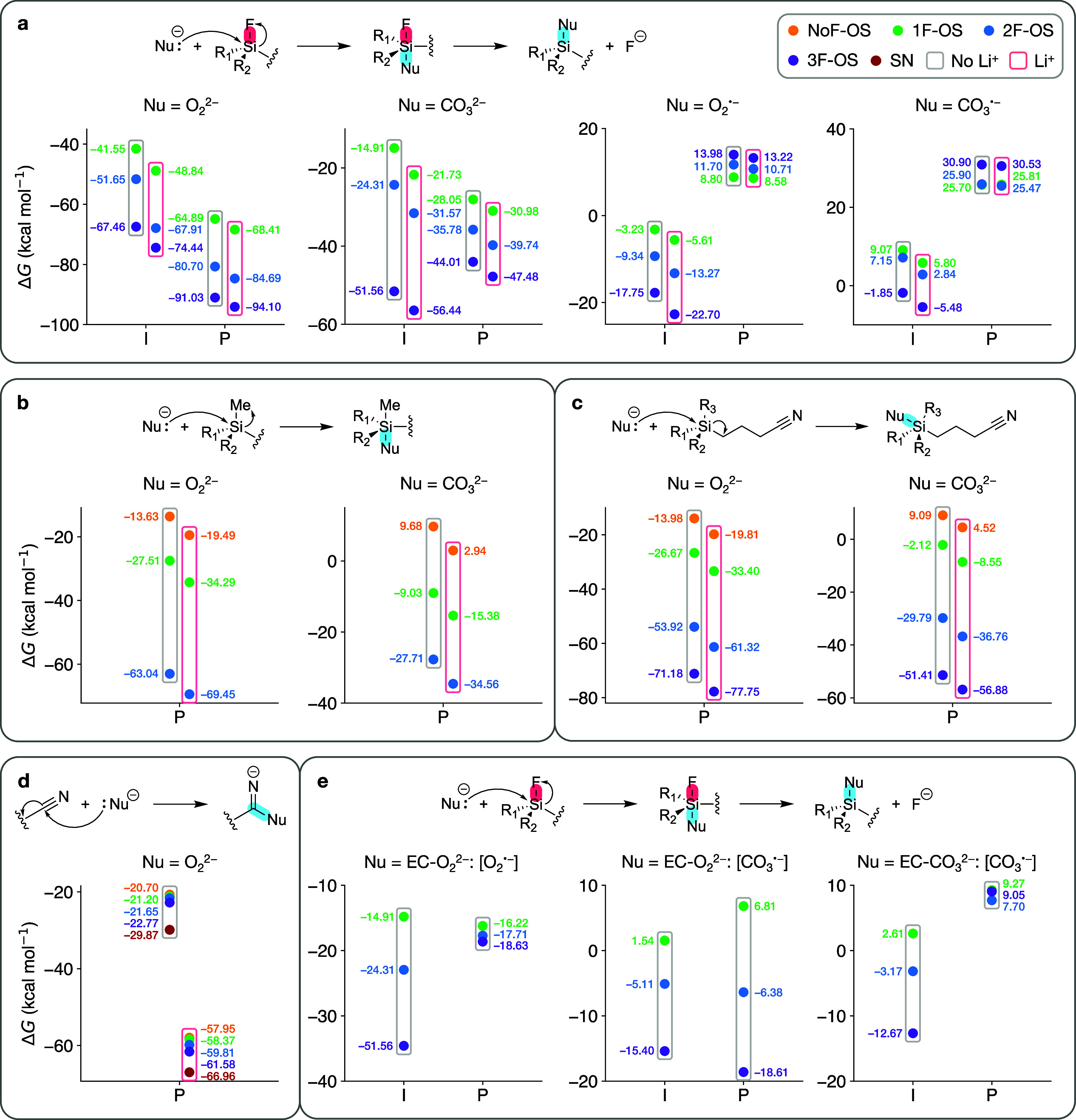
Relative free energies of the intermediate states (I) and products (P) of various chemical reactions between noncoordinated (gray-boxed)/Li^+^-coordinated (pink-boxed) OS additives and various nucleophiles. (a) Bimolecular nucleophilic substitution (S_N_2) via backside attack of an F atom attached to Si. (b) Nucleophilic addition (A_N_) via backside attack of a methyl group attached to Si. (c) A_N_ via backside attack of the −(CH_2_)_3_–C≡N moiety attached to Si. (d) A_N_ via attack of the cyano-C. (e) Same as (a), with the nucleophile being the [O_2_^•–^]/[CO_3_^•–^] part of ethylene carbonate oxide ion and the [CO_3_^•–^] part of ethylene dicarbonate ion. Nonreacting additive moieties are shown as squiggly lines in the molecular structures.

Figure 6a shows the free energy profiles of the S_N_2@Si reactions between various nucleophiles and OS, initiated by a backside attack of the fluorine in the silyl group. In this mechanism, the reactants first form an intermediate state with a pentacoordinate Si, which subsequently results in the expulsion of an F^–^ ion from the OS molecule. (Optimized geometries are shown in Figures S6 and S7.) This pathway is supported experimentally by the NMR spectroscopy results in a recent study involving KO_2_+OS solutions.^35^ Evidently, the relative free energy of the intermediate state (Δ*G*_int_) decreases with increasing degree of OS fluorination, contrary to the trends of the oxidation potentials. Curiously, we observe that the relative stability of the intermediate state and the products varies considerably across different nucleophiles. Here, Δ*G*_int_ follows a similar trend as in the EC ring-opening reactions. Specifically, (1) OS most efficiently scavenges peroxide (O_2_^2–^), followed by carbonate ion (CO_3_^2–^) and superoxide (O_2_^•–^); and (2) Li^+^ coordination of the OS at the cyano-N site lowers the free energies, although the effect is relatively weaker than that of fluorination.

Upon further inspection of Figure 6a, several notable features of these S_N_2@Si reactions stand out. First, unlike in S_N_2@C where the intermediate states are saddle points, the intermediate states in S_N_2@Si are local energy minima for all four nucleophiles, as confirmed by the absence of imaginary-frequency vibrational modes. This observation is consistent with previous studies^63,64^ attributing this behavior to the reduced steric repulsion of the substituents around the Si atom, suggesting that the relatively large size of Si is crucial in facilitating this reaction. Second, the structural stability of the intermediates varies considerably with the nucleophilicity of the substituting species. Precisely, for OS-O_2_^2–^, the intermediate states are positioned along the shoulders of their respective potential energy surfaces, as F^–^ readily dissociates from Si with minimal perturbation. In contrast, the intermediate states for the other three nucleophiles reside in local energy minima. Lastly, the intermediates become more stable relative to the products as the strength of the nucleophile decreases in the order O_2_^2**–**^ > CO_3_^2–^ > O_2_^•–^ > CO_3_^•–^. Interestingly, in the case of O_2_^•–^ and CO_3_^•–^, the energetic trends of the products are mostly reversed, with trifluorinated species having the highest Δ*G*. This observation indicates that the impact of the relatively unfavorable interactions between the weaker nucleophiles and Si outweighs that between Si and F^–^.

Since the silyl-Si in OS molecules is electrophilic, it can, in principle, attract nucleophiles from various directions. Depending on the direction of approach, the leaving group could be an F atom, a methyl group (−CH_3_), or the –(CH_2_)_3_–C≡N moiety. However, the latter two are unstable as anions and, therefore, unlikely to be separated from Si. Instead, these nucleophilic addition (A_N_) reactions typically form stable molecular complexes with pentacoordinate Si as the final products (optimized geometries are shown in Figures S8 and S9). Figures 6b,c show the energetics of such reactions. Compared with the F-substitution S_N_2 reactions, the reaction free energies are generally higher, suggesting that these pathways are less favorable. This is further supported by the observation that, with a few exceptions (Table S3), neither O_2_^•–^ nor CO_3_^•–^ can stably bind to Si through these mechanisms. In these A_N_@Si reactions, Li^+^ coordination and fluorination of OS induce similar energy-lowering effects as in the F-substitution reactions. These findings strongly suggest that a higher degree of fluorination increases the *likelihood of occurrence* for the energetically preferred F-substitution reactions, while lowering the reaction free energies for all the nucleophilic reaction pathways alike. We propose that these dual effects are the key contributing factors to the increased nucleophile-scavenging efficiency of fluorinated OS additives.

An alternative nucleophilic addition reaction may occur at the cyano-C of the additive molecules containing the cyano group(s). Figure 6d shows the energetics of this mechanism for both the OS molecules and succinonitrile (SN). It is found that, aside from peroxide, none of the other three nucleophiles bind favorably with cyano-C (with the exceptions shown in Table S3), making this pathway the least favorable among the chemical reaction mechanisms considered. Nevertheless, for peroxide alone, the reaction free energies with pure additive molecules are lower than –20 kcal mol^–1^, and Li^+^ coordination further reduces the reaction free energies by approximately 40 kcal mol^–1^ for all the additives. (Optimized geometries are shown in Figure S10.) This considerable reduction of free energy compared to the other pathways is attributed to the strong interaction between Li^+^ and the nucleophile. Notably, SN exhibits the lowest Δ*G*, though the differences in the reaction energies for various additives are minor (within 10 kcal mol^–1^). Since cyano-C nucleophilic addition is only viable for peroxide and not for the other nucleophiles, it becomes clear that the OS molecules are more efficient at scavenging gas-inducing nucleophiles compared to SN. This is due to the fact that silyl groups can bind favorably with multiple nucleophiles through various pathways described above.

**Figure 7:**
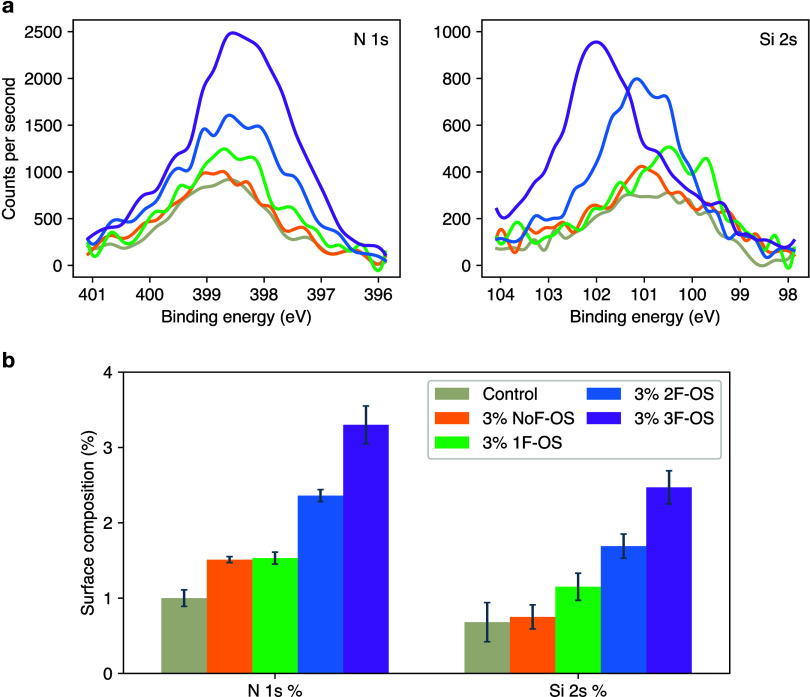
XPS surface analysis of NMC811 cathodes after formation in SC-NMC811/Gr pouch cells for control 1 and 3% NoF-OS/1F-OS/2F-OS/3F-OS electrolytes: (a) N 1s and Si 2s spectral regions; (b) percent composition of nitrogen and silicon out of all surface elements.

As discussed in the previous section, EC can form linear derivative species via S_N_2-type ring-opening reactions with negatively charged oxygen or CO_3_^2**–**^. The resulting [O_2_^•–^] and [CO_3_^•–^] moieties can also act as nucleophiles in the F-substitution S_N_2@Si reactions with OS molecules. Such reactions would lead to oligomers containing both EC and OS moieties. (Optimized geometries are shown in Figures S11 and S12.) Figure 6e shows the energetics of these reactions, which exhibit qualitative differences compared to the corresponding reactions with individual O_2_^•–^ and CO_3_^•–^ ions. The intermediate states are thermodynamically favorable, with the exception of 1F-OS in the [CO_3_^•–^] attack pathway. In terms of the [O_2_^•–^] attack mechanism, the F^–^-expulsion step is exergonic for 1F-OS but endergonic for 2F-OS and 3F-OS, while the opposite trend is observed for the [CO_3_^•–^] attack mechanism. Analogous to other pathways, we expect that Li^+^ coordination of OS would uniformly lower the free energies of these reactions. Each [EC-O_2_]^2–^ ion contains both moieties, suggesting this mechanism could potentially serve as initial steps leading to oligomers cross-linking [EC-O_2_]^2–^ and OS units. A similar pathway is found for ethylene dicarbonate ion, [EC–CO_3_]^2–^, formed from the EC+CO_3_^2–^ reaction. Such oligomeric products could subsequently form a protective layer on the cathode and help suppress further oxidative decomposition of solvents. This mechanism is qualitatively consistent with the surface analysis results of NMC811 cathodes after formation (Figure 7). Here, the greater intensities of Si 2s and N 1s spectra and the increased surface binding energy of Si for higher-fluorinated OS (Figure 7a) are indirect evidence of their increasing tendency of incorporation in the cathode-electrolyte interphase (CEI) layer. This trend is also directly reflected in the surface concentration of Si and N (Figure 7b), where an increased surface incorporation for higher-fluorinated OS is observed.

## Conclusions

In this combined computational and experimental study, we explore the multifaceted roles of organosilicon additives in suppressing gas evolution in liquid LIB electrolytes. The uniform gas-reducing functionalities of the OS additives are confirmed by pouch cell volume measurements and gas chromatography–mass spectrometry (GC-MS). Experimental results further indicate that these compounds are particularly effective at inhibiting CO_2_ generation regardless of source, with a positive correlation between OS’s degree of fluorination and their potency. Classical MD simulations reveal that OS additives preferentially replace EC and EMC in the first solvation shell of Li^+^ in a 1 M LiPF_6_ 1:1:1%vol EC:EMC:DEC mixture. In particular, we expect a majority (>85%) of the OS additives to bind with Li^+^, with a minimal negative effect on the electrolytes’ ionic conductivity when added in a small amount (<3%).

Through extensive DFT calculations, we find multiple plausible routes for CO_2_ generation in LIB electrolytes across different voltages. EC decomposition via S_N_2-type nucleophilic attack by anionic oxygen species (O_2_^2–^, O_2_^•–^) is favored at voltages higher than the onset potential of oxygen release from the metal oxide cathode (∼4.3 V vs Li^+^/Li), while EC reaction with carbonate ion (CO_3_^2–^) and direct electro-oxidation of Li_2_CO_3_ are favored at lower voltages. We observe that the oxidation potentials of OS and their complexes substantially exceed the normal operating range of NMC811 cathodes (>4.4 V vs Li^+^/Li), highlighting the OS compounds’ electro-oxidative stability while ruling out direct electro-oxidation as a feasible explanation of their gas reduction properties. More importantly, we discover that OS additives function through a dual chemical mechanism: (1) scavenging anionic oxygen species and carbonate ions, and (2) oligomerization with ring-opened EC derivatives such as ethylene carbonate oxide ion and ethylene dicarbonate ion. The boosted gas-reduction potency of fluorinated OS is attributed to both the increased likelihood of occurrence for the energetically preferred F-substitution S_N_2@Si reactions and the universal lowering of reaction free energies for various pathways. These findings are qualitatively corroborated by the XPS surface analysis results indicating the incorporation of OS components in the cathode surface. Finally, we show that Li^+^-coordinated OS lowers the free energies for most pathways, underscoring the importance of Li^+^ coordination in enhancing the gas-reducing potency of OS additives. In summary, our results provide a foundation for further exploration of the beneficial roles of organosilicon additives in improving both the performance and safety of LIBs.

## Supporting Information Available

Materials and methods; supplementary experimental and computational results including species-resolved gas volume measurements data, radial distribution functions, calculated ionic conductivities, optimized molecular geometries for various chemical/electrochemical reactions, OS partial charges, Li^+^ binding energies, and information on additional exergonic reactions (PDF)
