# Supplementary material for: Elucidating Gas Reduction Effects of Organosilicon Additives in Lithium-Ion Batteries
Source: J Am Chem Soc. 2025 Feb 26;147(10):8841–51. doi: 10.1021/jacs.5c00402 (PMC11912472; doi:10.1021/jacs.5c00402)
Supplement: Supplementary file 1 — ja5c00402_si_001.pdf [file ja5c00402_si_001.pdf]

Supplementary Information

Elucidating Gas Reduction Effects of Organosilicon Additives in Lithium-Ion Batteries

Jingyang Wang[a], Sarah L. Guillot[b], Monica L. Usrey[b], Tingzheng Hou*[,c,d] , Kristin A. Persson*[,a,c]

[a] Materials Division, Lawrence Berkeley National Laboratory, Berkeley, California 94720, United States
[b] Silatronix, Inc. Madison, Wisconsin 53704, United States
[c] Department of Materials Science and Engineering, University of California Berkeley, Berkeley, California 94720, United States
[d] Institute of Materials Research, Tsinghua Shenzhen International Graduate School, Tsinghua University, Shenzhen, Guangdong 518055, China

E-mail: tingzhenghou@sz.tsinghua.edu.cn; kristinpersson@berkeley.edu

Contents

Materials and Methods....................2-4
Figure S1-S12....................5-14
Table S1-S3....................14-15
References....................16-17

Materials and Methods

Molecular Dynamics Simulations

The classical molecular dynamics (MD) simulations presented in this work were conducted with the Large Scale Atomic/Molecular Massively Parallel Simulator (LAMMPS)[1] package. The Optimized Potentials for Liquid Simulations All Atom (OPLS-AA) force field[2] was employed to model intra- and inter-molecular interactions. The equilibrium bond, angle, dihedral parameters, and nonbonded Lennard-Jones parameters for the solvents involved were obtained from the LigParGen server,[3] while those of ions were obtained from Sambasivarao and Acevedo.[4] The coulombic interactions were evaluated with the restrained electrostatic potential (RESP) partial charges[5] calculated directly from DFT, performed using the B3LYP functional[6–9] and the aug-cc-pvdz basis set,[10–12] as implemented in the Gaussian 16 package. These charges were calculated in both vacuum and implicit solvent under the polarizable continuum model (PCM)[13] with the solvent parameters set to those of acetone (dielectric constant $\varepsilon = 20.7$).

The systems studied in this work consist of various concentrations of additives solvated in a mixture of solvents and salts, designed to replicate the experimental conditions. The force field parameters for the OS molecules were benchmarked using model systems containing either pure OS or an OS + $LiPF_6$ mixture. The simulated solutions consist of 145 $LiPF_6$, 685 EC, 377 DEC, and 438 EMC molecules, along with 0/8/16/40 OS molecules, corresponding respectively to the experimental additive concentrations of 0%, 1%, 2%, and 5%. Molecules were initially packed randomly in a cubic box of dimensions 70×70×70 $Å^3$, with periodic boundary conditions in all three directions. The initial configuration was minimized using conjugated-gradient optimization. The systems were equilibrated for 3 ns in the isothermal-isobaric ensemble (constant NPT) using the Parrinello–Rahman barostat[14] to maintain a pressure of 1 bar and a temperature of 298 K with a time constant of 1ps. Production runs of 20 ns were conducted in the canonical ensemble (NVT) under Nose-Hoover thermostats[15,16] with a time constant of 1 ps at 298 K. Self-diffusion coefficients were computed from the MD trajectories by analyzing the mean square displacement (MSD) over time using the Stokes−Einstein relation:

$$D = \frac{1}{6} \lim_{t \to \infty} \frac{d}{dt} \langle (\delta r)^2 \rangle \qquad [1]$$

The ionic conductivity (σ) was obtained according to the Green–Kubo relation using the following expression:

$$\sigma = \frac{1}{6 k_B T V} \lim_{t \to \infty} \frac{d}{dt} \langle \sum_{i=1}^{N} \sum_{j=1}^{N} q_i q_j [\vec{r}_i(t) - \vec{r}_i(0)] \cdot [\vec{r}_j(t) - \vec{r}_j(0)] \rangle, \qquad [2]$$

where $k_B T$ is the thermal energy, V is volume, q_i is the charge of species i, and $\vec{r}_i(t)$ is the coordinates of species *i* at time *t*.

Density Functional Theory Calculations

The molecular density functional theory (DFT) calculations were conducted with the Q-Chem quantum chemistry package (version 6), with the ωB97M-V functional[17] and the def2-SVPD basis set.[18] Randomly selected optimized transition states and products were

reevaluated with the def2-TZVPPD basis set; their free energy differences thus obtained differ from the def2-SVPD results by less than 0.3 kcal/mol, confirming the sufficient accuracy of the def2-SVPD basis set. The solvation effect is implicitly included within the "solvent model with density" (SMD) scheme,[19] with the relevant parameters set to be those of acetone (dielectric constant $\varepsilon = 20.7$). Geometry optimization using the eigenvector-following algorithm was employed to find the structure and energy of stable single molecules, reactants, and products, while transition state optimization was used to find those of transition states. The energy and force convergence thresholds were 1×10^{-6} Hartree and 3×10^{-4} Hartree/Bohr, respectively. Stable structures were identified if either no imaginary frequency is present, or only one small imaginary frequency ($|\nu_{\mathrm{min}}| < 50\mathrm{cm}^{-1}$) is found. Transition state structures were identified if one single large imaginary frequency was found.

The DFT-calculated relative free energies provide a quantitative analysis of the relative stability of the relevant structure compared to the reference, hence is correlated with the likelihood of chemical or electrochemical reactions. The former was assessed using the thermodynamic free energy difference $\Delta G = G_{\mathrm{product}} - G_{\mathrm{reactant}}$, or the kinetic free energy difference $\Delta G^{\ddagger} = G^{\ddagger} - G_{\mathrm{reactant}}$, where $G^{\ddagger}$ is the absolute free energy of the transition state. The latter was assessed using the oxidation potential in the Li^+/Li scale, evaluated as $E_{\mathrm{oxd}} = (G_+ - G_0)/F - 1.4\mathrm{V}$, where G_+ and G_0 respectively refer to the free energy of the oxidized and unoxidized molecule at 298.15K, and F is Faraday's constant. The basis set superposition error (BSSE) was ignored in this work.

Electrolyte Preparation

All carbonate solvents and $LiPF_6$ were purchased electrochemical grade from Gotion and SoulBrain (water content < 20 ppm). Vinylene carbonate (VC) was purchased from BASF. The organosilicon materials (NoF-OS, 1F-OS, 2F-OS, and 3F-OS) were Silatronix® electrochemical grade (> 99.8% purity, < 20 ppm water). The control electrolyte was a blend of EC, ethyl methyl carbonate (EMC), and diethyl carbonate (DEC) in the ratio EC/EMC/DEC 1/1/1 by volume, with 0.5 vol% VC (Control 1) or 1 vol% VC (Control 2) and 1 M $LiPF_6$. The organosilicon materials were added to the control 3% by volume of the solvent blend. All electrolytes were prepared and stored in an argon glove box. In an experiment used to determine the origin of CO_2, all electrolytes (Control 2, 3% 1F-OS, 1% 2F-OS, 0.75% 3F-OS) were formulated with 32.4-33.2 vol% of the EC replaced by $^{13}C_3$-EC.

Pouch Cell Testing

Dry, pre-packaged 230 mAh 4.3 V single crystal NMC811/artificial graphite multilayer pouch cells were purchased from LiFun. The cells were dried under vacuum at 60 °C for 48 hours and then filled with 1 mL of electrolyte. Three cells were used per electrolyte formulation. The pouch cells underwent 45°C formation followed by charging to 4.3 V and storing at OCV in a 60 °C oven for 4 weeks. The formation procedure was (1) C/20 charge to 4.0 V; (2) pause and degas in argon glovebox; (3) C/10 charge/discharge, 2.75-4.30 V; (4) C/5 charge/discharge, 2.75-4.3 V; (5) C/5 charge to 3.8 V (50% SOC). Gas volumes of the pouch cells were measured by the Archimedes method, as previously established in Aiken *et al.*[20]

Gas Chromatography-Mass Spectrometry

Gas generated during 60 °C storage was extracted from the pouch cells after storage using a gas-tight syringe (Hamilton 1710N—100 µl) through an adhesive foam septum (Quantek Instruments). The gas from non-^{13}C labeled electrolytes was injected into an Agilent 6890 N Gas Chromatograph (TCD detector) equipped with a dual column system: HP-Plot Q (30 m length, 0.32 mm ID, 20.00 µm film thickness, 7-inch cage) and HP-PLOT Molesieve (20 m length, 0.32 mm ID, 25.00 µm film thickness, 7-inch cage). The dual column system allowed the separation of permanent gases (N_2, O_2, H_2) and other gaseous components (CO_2, CO, methane, ethane, ethylene) and has been described previously for full analysis of battery gas compositions. The system was calibrated using several standard gas mixtures to allow accurate quantitative analysis for H_2, CO_2, CO, N_2, O_2, methane, ethane, ethylene, propane, and propylene. For all calibrated species, 5% and 1% mixtures in argon (custom, Praxair) were used. Quantities of propane, propylene, and ethylene were negligible and therefore are not shown in figures reporting gas compositions. The GC program was optimized to provide separation of the required gaseous species: 20 °C min^{-1} ramp from 50 °C to 70 °C, −20 °C min^{-1} ramp from 70 °C to 50 °C, 35 °C min^{-1} ramp from 50 °C to 250 °C, and 8 min hold at 250 °C. Argon was used as the carrier gas (29.7 psi pressure). The gas from the electrolytes containing $^{13}C_3$-EC was injected into a Shimadzu Ultra Gas chromatograph mass spectrometer with an HP-PLOT Q column (30 m length, 0.32 mm ID 20 µm film thickness). The GC program used a 20 °C min^{-1} ramp from 45 °C to 155 °C and 1.5 min hold at 155 °C with 1.35 mL/min column flow and 100:1 split ratio. The MS program used single ion monitoring acquisition mode (m/z 44 and 45 used to analyze $^{12}CO_2$ and $^{13}CO_2$) with 250 °C interface temperature and 200 °C ion source temperature.

X-ray photoelectron spectroscopy surface analysis

For surface analysis, the cells were disassembled after formation (at 50% SOC) in an argon glove box. Pieces of the anode and cathode were excised, rinsed three times with electrochemical grade dimethyl carbonate purchased from Sigma Aldrich and SoulBrain, and dried at room temperature overnight under vacuum (~50 mTorr). The samples were loaded into an air-free XPS sample holder (Vacuum Transfer Module, Thermo Fisher) to prevent contamination during transport to and loading into a Thermo k-alpha X-ray photoelectron spectrometer with Al source (Nanoscale Imaging and Analysis Center, University of Wisconsin-Madison). Spectra were collected with a 200 µm spot size and 45° detection angle with a flood gun on (samples were attached to the sample holder with double-sided silicon-free tape). Survey scans (0–1400 eV, 5 scans) were collected at 1 keV. XPS spectra were processed with CasaXPS (version 2.3.19), and multiplex spectra were referenced to the adventitious carbon peak at 284.8 eV. After defining quantification regions for every element observed in the survey spectra, atomic percent concentrations of each element of interest were calculated by CasaXPS as a function of the total surface elemental composition, using the Scofield response factors, which are appropriate for the XPS instrument used in this study.

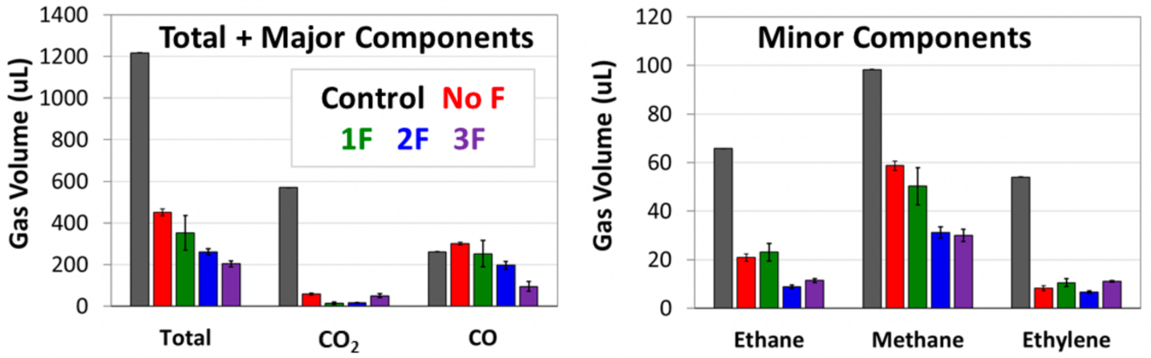

Figure S1. Species-resolved gas volume increase after 4 weeks of storage at 60 °C relative to after formation in 4.3 V SC-NMC811/Gr pouch cells with control 1 electrolyte.

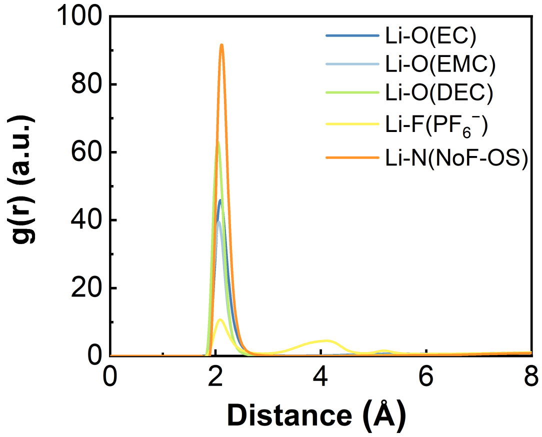

Figure S2. The radial distribution functions (RDFs) of Li-O(EC), Li-O(EMC), Li-O(DEC), Li-F(PF_6^-), Li-N(NoF-OS) pairs in 1 M $LiPF_6$ 1:1:1 %vol EC:EMC:DEC with 5% OS additive using PCM charge models.

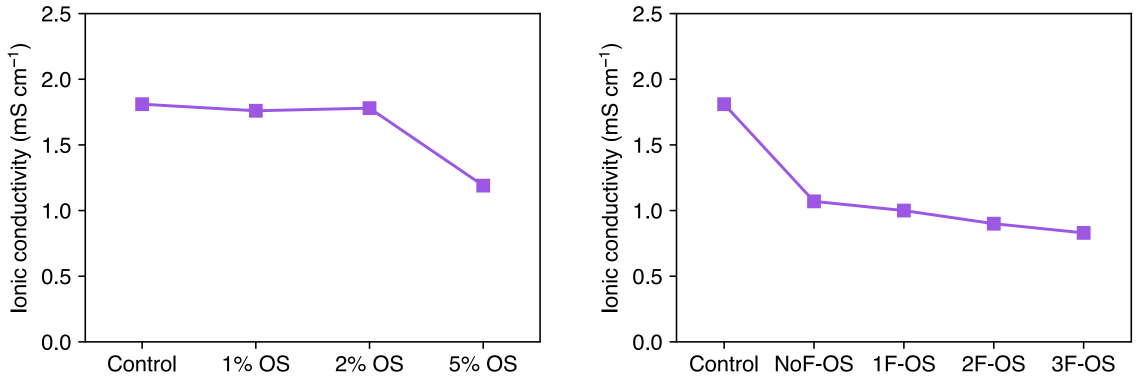

Figure S3. The calculated ionic conductivity in 1 M $LiPF_6$ 1:1:1 (%vol) EC:EMC:DEC solutions. (Left) control and 1/2/5% NoF-OS electrolytes; (right) control and 5% NoF-OS/1F-OS/2F-OS/3F-OS electrolytes.

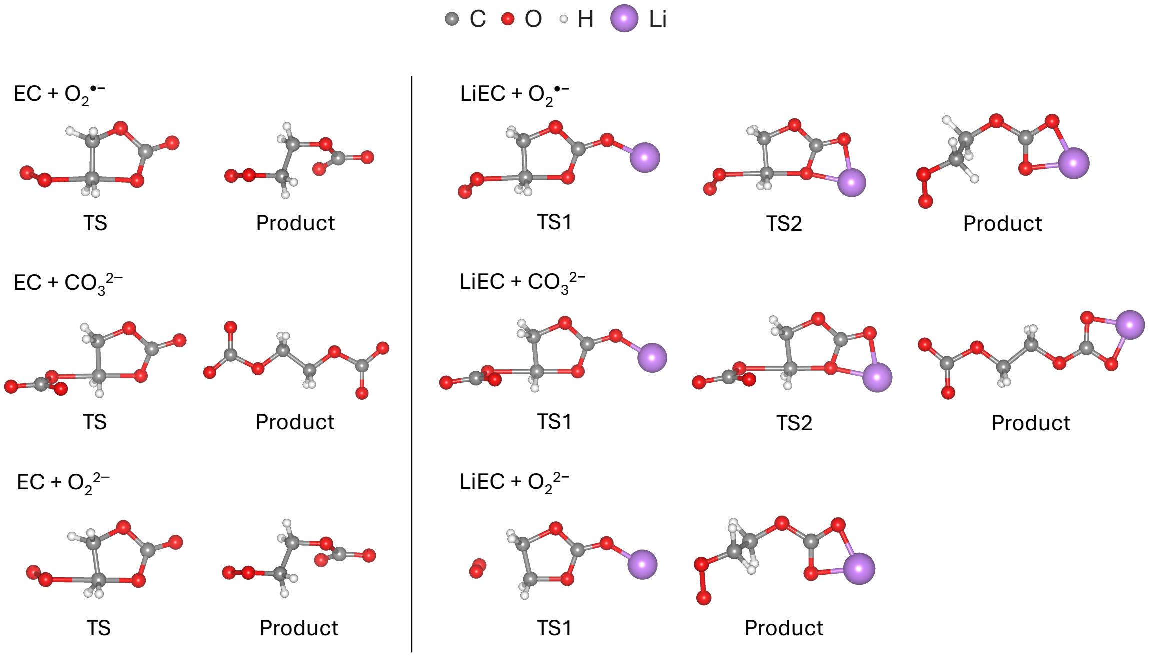

Figure S4. Optimized geometries of the intermediate and product states of S_N2@C between noncoordinated/Li^+-coordinated EC and anionic oxygen species ($O_2^{\bullet-}$/CO_3^{2-}/O_2^{2-}).

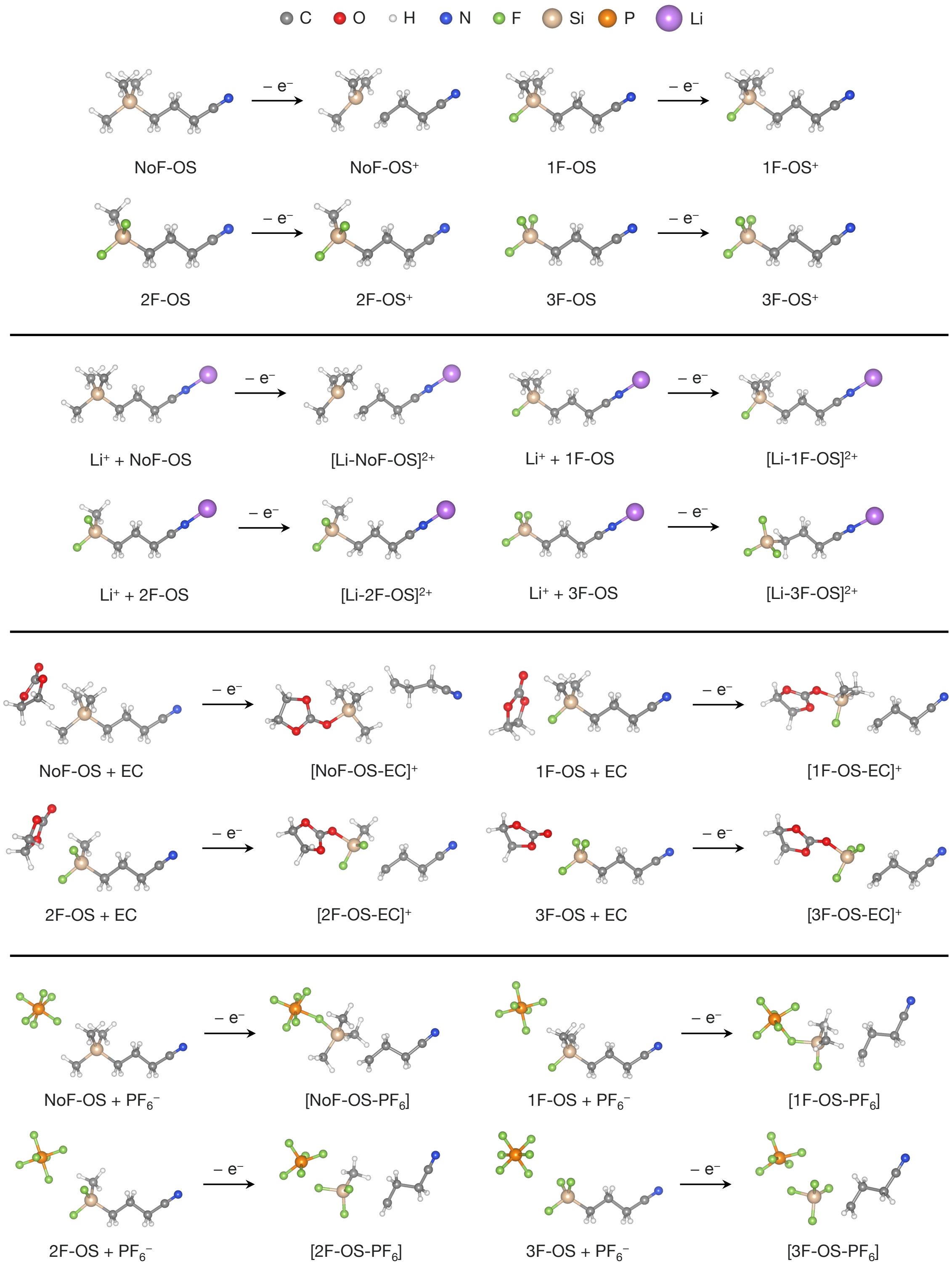

Figure S5. Optimized geometries of OS, Li-OS, OS-EC, and OS-PF_6 (OS = NoF-OS/1F-OS/2F-OS/3F-OS), in their native and oxidated states.

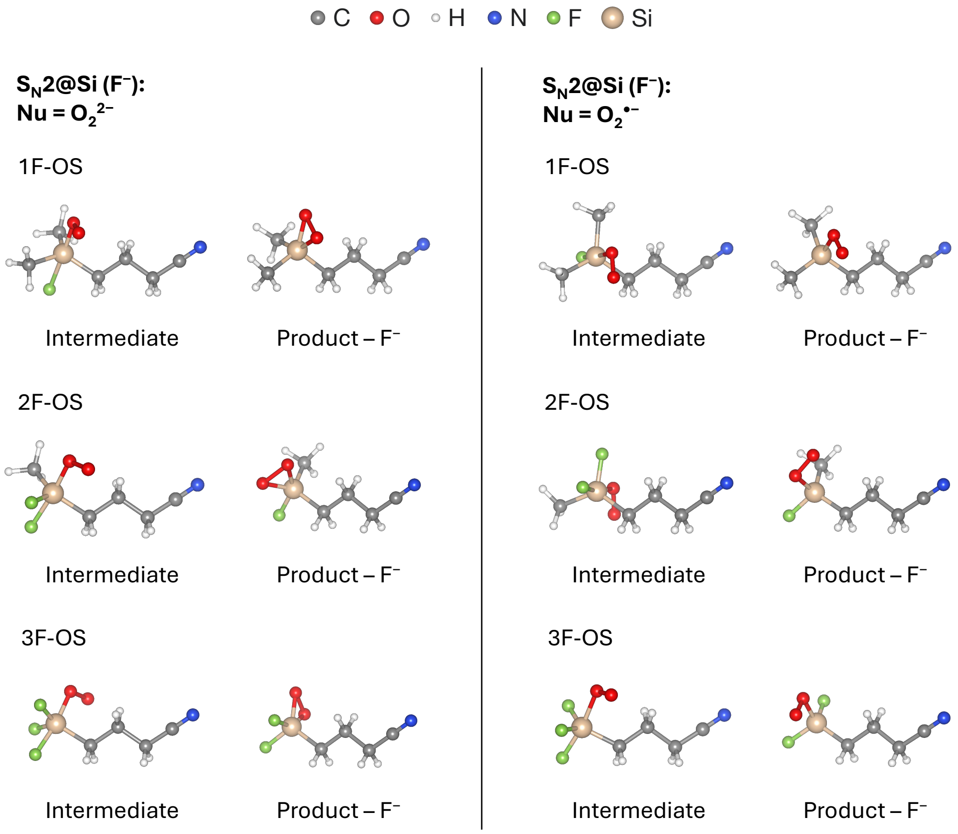

Figure S6. Optimized geometries of the intermediate and product states of S_N2@Si (F) reactions between OS and anionic oxygen species (O_2^{2-}/$O_2^{\bullet-}$).

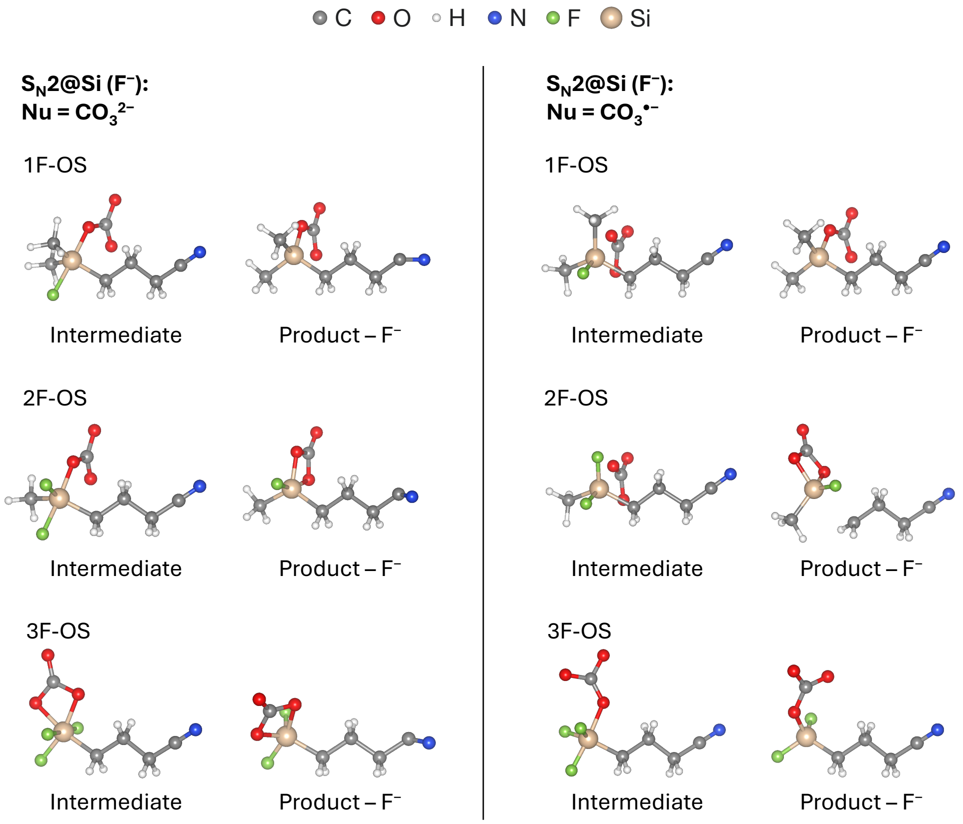

Figure S7. Optimized geometries of the intermediate and product states of S_N2@Si (F) reactions between OS and carbonate ions (CO_3^{2-}/$CO_3^{\bullet-}$).

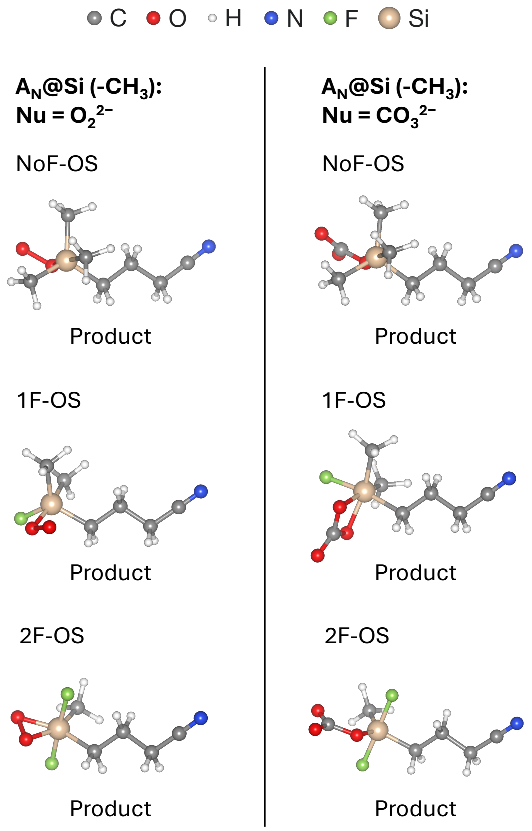

Figure S8. Optimized geometries of the product states of A_N@Si (−CH_3) reactions between OS and peroxide (O_2^{2-}) / carbonate ion (CO_3^{2-}).

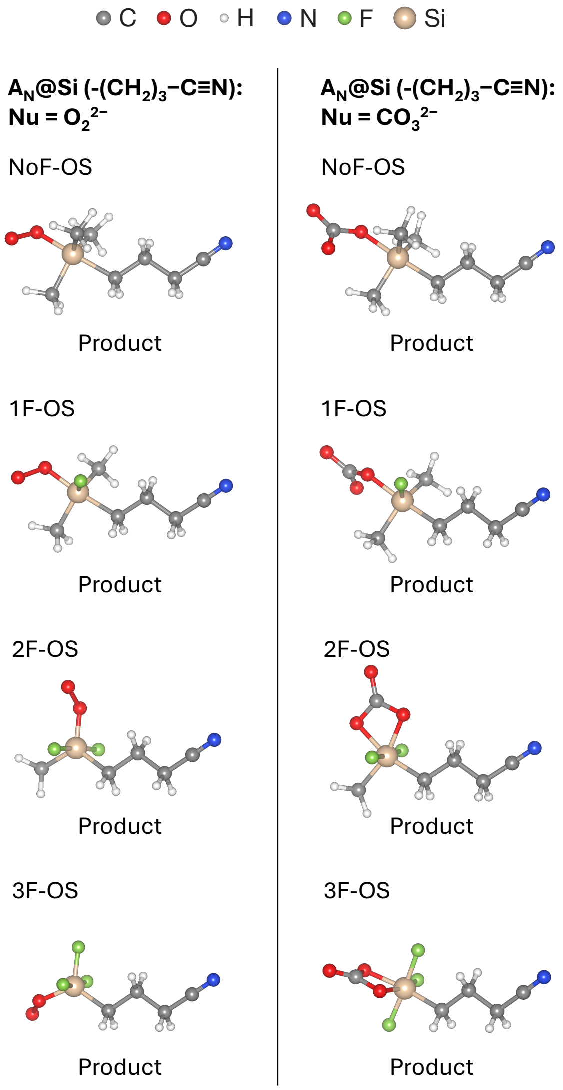

Figure S9. Optimized geometries of the product states of A$_N$@Si (−(CH$_2$)$_3$−C≡N) reactions between OS and peroxide (O_2^{2-}) / carbonate ion (CO_3^{2-}).

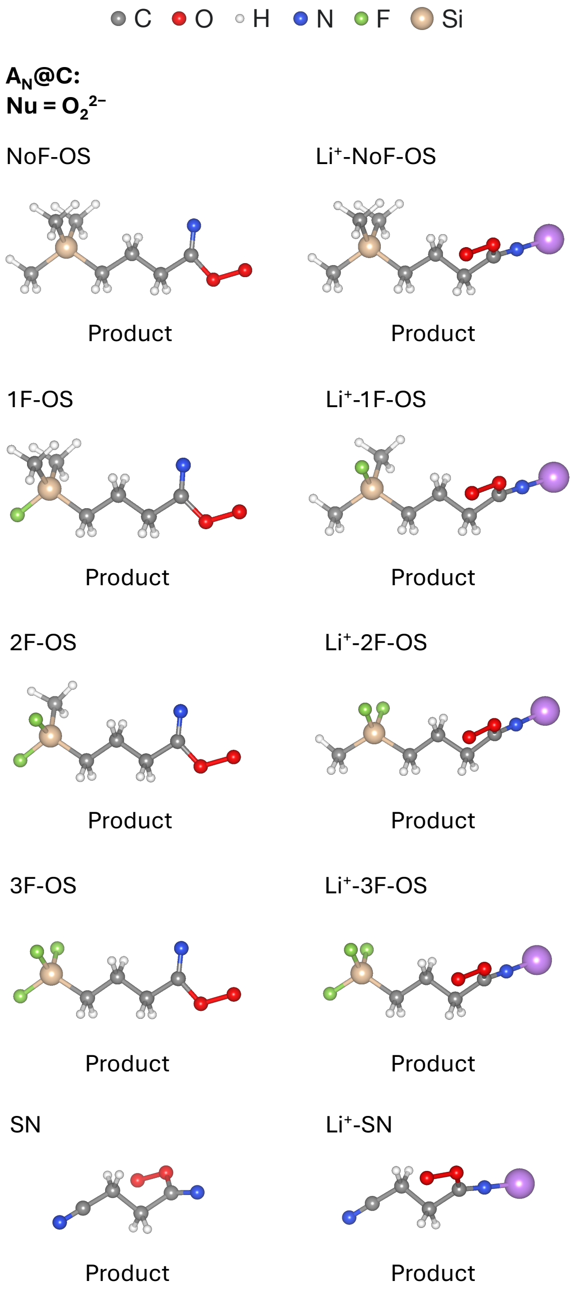

Figure S10. Optimized geometries of the intermediate and product states of A_N@C reactions between pure/lithiated OS and peroxide (O_2^{2-}).

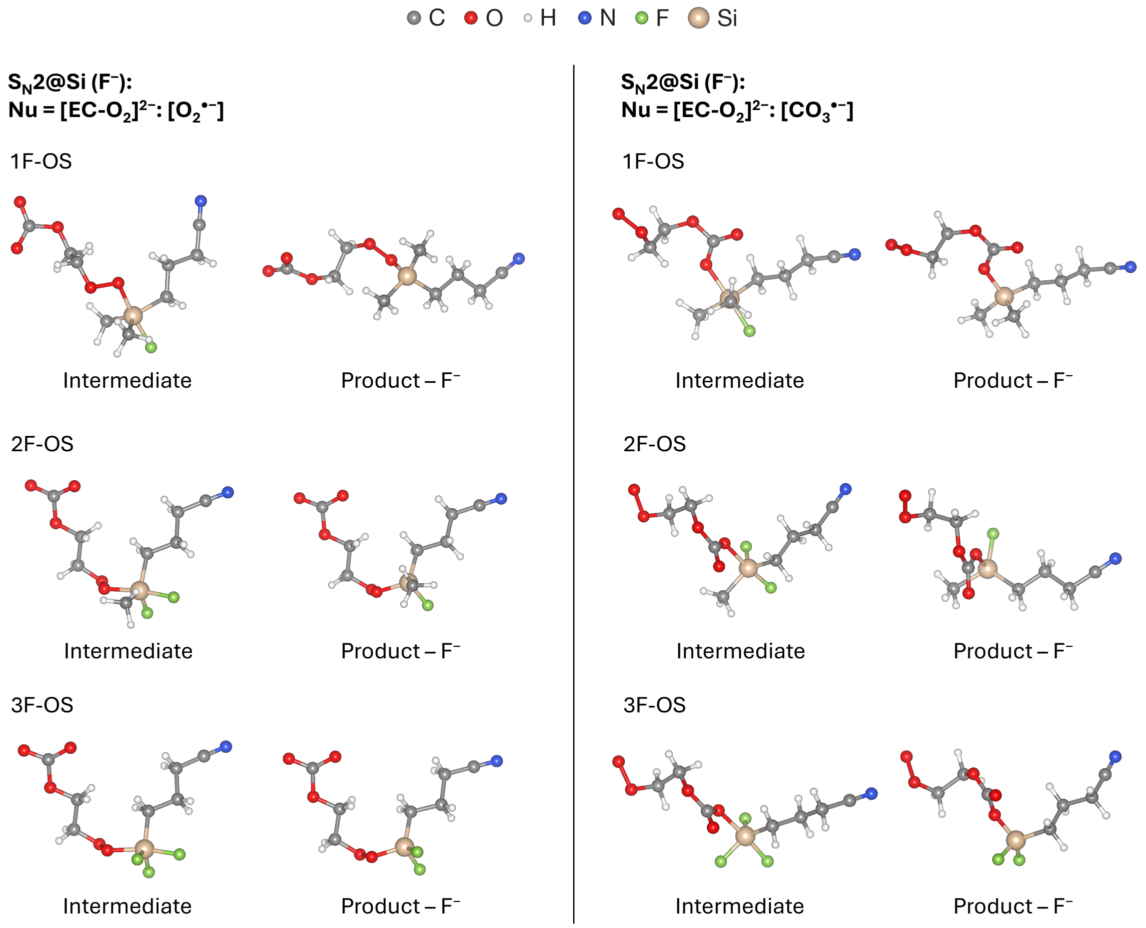

Figure S11. Optimized geometries of the intermediate and product states of A_N@Si (F) reactions between OS and $[EC\text{-}O_2]^{2-}$.

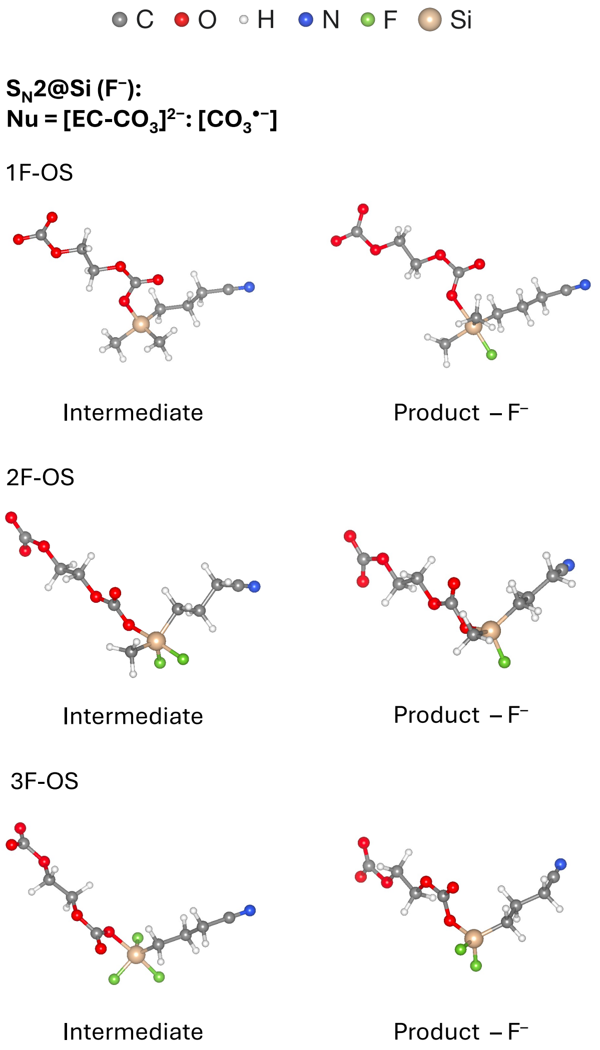

Figure S12. Optimized geometries of the intermediate and product states of A_N@Si (F) reactions between OS and [EC-CO_3]$^{2-}$.

Additive	N charge (PCM)	C charge (PCM)
NoF-OS	-0.576	0.394
1F-OS	-0.573	0.397
2F-OS	-0.564	0.383
3F-OS	-0.555	0.373

Table S1. Partial charges of selected atoms in OS additives, calculated in a PCM solvation model of acetone (dielectric constant dielectric constant $\varepsilon = 20.7$).

Additive	Li^+ binding energy (kcal/mol)
EC	-16.50
NoF-OS	-16.53
1F-OS	-15.93
2F-OS	-15.23
3F-OS	-14.42

Table S2. The binding energy of the relevant species in this work (EC, OS additives) to Li^+ ion.

Mechanism	Species	Nucleophile	ΔG (kcal/mol)
A_N@Si ($-CH_3$)	2F-OS	$O_2^{\bullet -}$	-5.13
	Li^+-2F-OS	$O_2^{\bullet -}$	-9.19
A_N@Si ($-(CH_2)_3-C \equiv N$)	2F-OS	$O_2^{\bullet -}$	-5.16
	Li^+-2F-OS	$O_2^{\bullet -}$	-4.94
	3F-OS	$O_2^{\bullet -}$	-12.05
	Li^+-3F-OS	$O_2^{\bullet -}$	-15.77
	3F-OS	$CO_3^{\bullet -}$	-0.18
	Li^+-3F-OS	$CO_3^{\bullet -}$	-4.51

Table S3. Exceptional cases of exergonic reactions between additive and nucleophiles.

References

(1) Thompson, A. P.; Aktulga, H. M.; Berger, R.; Bolintineanu, D. S.; Brown, W. M.; Crozier, P. S.; In 'T Veld, P. J.; Kohlmeyer, A.; Moore, S. G.; Nguyen, T. D.; Shan, R.; Stevens, M. J.; Tranchida, J.; Trott, C.; Plimpton, S. J. LAMMPS - a Flexible Simulation Tool for Particle-Based Materials Modeling at the Atomic, Meso, and Continuum Scales. *Comput. Phys. Commun.* **2022**, *271*, 108171.

(2) Jorgensen, W. L.; Maxwell, D. S.; Tirado-Rives, J. Development and Testing of the OPLS All-Atom Force Field on Conformational Energetics and Properties of Organic Liquids. *J. Am. Chem. Soc.* **1996**, *118* (45), 11225–11236.

(3) Dodda, L. S.; Cabeza de Vaca, I.; Tirado-Rives, J.; Jorgensen, W. L. LigParGen Web Server: An Automatic OPLS-AA Parameter Generator for Organic Ligands. *Nucleic Acids Res.* **2017**, *45* (W1), W331–W336.

(4) Sambasivarao, S. V.; Acevedo, O. Development of OPLS-AA Force Field Parameters for 68 Unique Ionic Liquids. *J. Chem. Theory Comput.* **2009**, *5* (4), 1038–1050.

(5) Bayly, C. I.; Cieplak, P.; Cornell, W.; Kollman, P. A. A Well-Behaved Electrostatic Potential Based Method Using Charge Restraints for Deriving Atomic Charges: The RESP Model. *J. Phys. Chem.* **1993**, *97* (40), 10269–10280.

(6) Becke, A. D. Density-Functional Thermochemistry. III. The Role of Exact Exchange. *J. Chem. Phys.* **1993**, *98* (7), 5648–5652.

(7) Lee, C.; Yang, W.; Parr, R. G. Development of the Colle-Salvetti Correlation-Energy Formula into a Functional of the Electron Density. *Phys. Rev. B* **1988**, *37* (2), 785–789.

(8) Vosko, S. H.; Wilk, L.; Nusair, M. Accurate Spin-Dependent Electron Liquid Correlation Energies for Local Spin Density Calculations: A Critical Analysis. *Can. J. Phys.* **1980**, *58* (8), 1200–1211.

(9) Stephens, P. J.; Devlin, F. J.; Chabalowski, C. F.; Frisch, M. J. Ab Initio Calculation of Vibrational Absorption and Circular Dichroism Spectra Using Density Functional Force Fields. *J. Phys. Chem.* **1994**, *98* (45), 11623–11627.

(10) Kendall, R. A.; Dunning, T. H.; Harrison, R. J. Electron Affinities of the First-Row Atoms Revisited. Systematic Basis Sets and Wave Functions. *J. Chem. Phys.* **1992**, *96* (9), 6796–6806.

(11) Woon, D. E.; Dunning, T. H. Gaussian Basis Sets for Use in Correlated Molecular Calculations. III. The Atoms Aluminum through Argon. *J. Chem. Phys.* **1993**, *98* (2), 1358–1371.

(12) Prascher, B. P.; Woon, D. E.; Peterson, K. A.; Dunning, T. H.; Wilson, A. K. Gaussian Basis Sets for Use in Correlated Molecular Calculations. VII. Valence, Core-Valence, and Scalar Relativistic Basis Sets for Li, Be, Na, and Mg. *Theor. Chem. Acc.* **2011**, *128* (1), 69–82.

(13) Miertuš, S.; Scrocco, E.; Tomasi, J. Electrostatic Interaction of a Solute with a Continuum. A Direct Utilizaion of AB Initio Molecular Potentials for the Prevision of Solvent Effects. *Chem. Phys.* **1981**, *55* (1), 117–129.

(14) Parrinello, M.; Rahman, A. Polymorphic Transitions in Single Crystals: A New Molecular Dynamics Method. *J. Appl. Phys.* **1981**, *52* (12), 7182–7190.

(15) Nosé, S. A Unified Formulation of the Constant Temperature Molecular Dynamics Methods. *J. Chem. Phys.* **1984**, *81* (1), 511–519.

(16) Hoover, W. G. Canonical Dynamics: Equilibrium Phase-Space Distributions. *Phys. Rev. A* **1985**, *31* (3), 1695–1697.

(17) Mardirossian, N.; Head-Gordon, M. ωB97M-V: A Combinatorially Optimized, Range-Separated Hybrid, Meta-GGA Density Functional with VV10 Nonlocal Correlation. *J. Chem. Phys.* **2016**, *144* (21), 214110.
(18) Rappoport, D.; Furche, F. Property-Optimized Gaussian Basis Sets for Molecular Response Calculations. *J. Chem. Phys.* **2010**, *133* (13), 134105.
(19) Marenich, A. V.; Cramer, C. J.; Truhlar, D. G. Universal Solvation Model Based on Solute Electron Density and on a Continuum Model of the Solvent Defined by the Bulk Dielectric Constant and Atomic Surface Tensions. *J. Phys. Chem. B* **2009**, *113* (18), 6378–6396.
(20) Aiken, C. P.; Xia, J.; Wang, D. Y.; Stevens, D. A.; Trussler, S.; Dahn, J. R. An Apparatus for the Study of In Situ Gas Evolution in Li-Ion Pouch Cells. *J. Electrochem. Soc.* **2014**, *161* (10), A1548–A1554.
